# suPAR as a marker of infection in acute kidney injury – a prospective observational study

**DOI:** 10.1186/s12882-018-0990-6

**Published:** 2018-08-02

**Authors:** Anna Hall, Siobhan Crichton, Matt Varrier, Danielle E. Bear, Marlies Ostermann

**Affiliations:** 1 Guy’s & St Thomas’ NHS Foundation Trust, Department of Critical Care, London, SE1 9RT UK; 2 MRC Clinical Trials Unit, University College London, London, WC2B 6NH UK; 3 King’s College London, Guy’s & St Thomas’ NHS Foundation Trust, Department of Critical Care, London, SE1 9RT UK; 4 Guy’s & St Thomas’ NHS Foundation Trust, Departments of Nutrition and Dietetics & Critical Care, London, SE1 9RT UK; 5 King’s College London, Guy’s and St Thomas’ Foundation Hospital, Department of Critical Care, London, SE1 7EH UK

**Keywords:** Acute kidney injury, suPAR, uPAR, Infection, CRP, Soluble urokinase-type plasminogen activator receptor

## Abstract

**Background:**

Soluble urokinase-type plasminogen activator receptor (suPAR) has emerged as a new sepsis biomarker. It is not known whether suPAR has a role in critically ill patients with severe acute kidney injury (AKI).

**Methods:**

Our main aims were to describe serial serum suPAR concentrations in patients with severe AKI, to investigate a potential association between suPAR and C-reactive protein (CRP), and to compare suPAR and CRP as diagnostic markers of infection in patients with AKI. Between April 2013 – April 2014, we recruited adult patients (≥18 years) with AKI KDIGO stage 2/3 admitted to a multidisciplinary Intensive Care Unit (ICU) in a University Hospital in UK. Serial serum suPAR and CRP concentrations were measured for 6 days. We compared the characteristics and serial suPAR and CRP concentrations of patients with and without an infection using Chi-squared, Fisher’s exact, t-test and Mann-Whitney tests as appropriate, and calculated the area under the receiver operating characteristics curve (AUC).

**Results:**

Data of 55 patients with AKI stage 2/3 were analysed (62% male; mean age 60.5) of whom 43 patients received continuous renal replacement therapy. suPAR was not detectable in effluent fluid.

There was no significant correlation between daily suPAR and CRP concentrations. In patients with an infection, suPAR results were significantly higher than in those without an infection across all time points; there was no significant difference in CRP levels between both groups. After exclusion of patients with an infection before or on day of admission to ICU, the AUC of suPAR for predicting an infection later was 0.62 (95% CI 0.43–0.80) compared to 0.50 (95% CI 0.29–0.71) for CRP.

**Conclusions:**

In critically ill patients with AKI stage 2/3, suPAR is a better marker of infection than CRP.

**Trial registration:**

The study was retrospectively registered on the ISRCTN registry on 25 November 2012 (ISRCTN88354940).

## Background

Acute kidney injury (AKI) affects > 50% of patients in the intensive care unit (ICU) [1]. It is associated with an increased risk of complications and mortality. Sepsis is particularly common but can be challenging to diagnose due to potentially misleading clinical signs and a limited number of confirmatory diagnostic tests in routine clinical practice [2].

Over 150 sepsis biomarkers have been identified [3]. C-reactive protein (CRP) is used mainly as a marker of inflammation. Measuring and charting CRP values can prove useful in determining disease progress or the effectiveness of treatments but it has limited ability to distinguish sepsis from other inflammatory conditions [3, 4]. Soluble urokinase-type plasminogen activator receptor (suPAR) is one of several newer sepsis markers that has been investigated in recent years [5–8]. SuPAR is the soluble form of the cell membrane-bound urokinase plasminogen activator receptor (uPAR) which is expressed on various cell types, including neutrophils, lymphocytes, monocytes, endothelial cells and tumour cells. After cleavage from the cell surface, suPAR is released into the blood and most human body fluids. When inflammatory cells are activated by cytokines, the expression of uPAR is up-regulated, thus increasing the serum levels of suPAR, too [9].

Several studies have shown higher systemic suPAR concentrations in critically ill patients compared to healthy controls, and in patients with infections compared to those without [5, 7, 8, 10]. However, a systematic review of relevant papers published until May 2011 reported that the diagnostic value of suPAR was low in critically ill patients with sepsis [11]. A subsequent meta-analysis in 2016 concluded that suPAR had a role as a biomarker for the diagnosis and prognosis of bacterial infections but was relatively ineffective for differentiating sepsis from systemic inflammation [12]. Both meta-analyses were limited by inclusion of heterogenous patient populations with varying acute and chronic illnesses. Whether there is a role for suPAR in critically ill patients with AKI is unknown.

## Methods

### Aims

The aims of our study were: a) to describe serial systemic suPAR values in patients with severe AKI, including patients on continuous renal replacement therapy (CRRT); b) to explore whether suPAR was removed during CRRT; c) to investigate a potential association between suPAR and CRP, and d) to compare suPAR and CRP as diagnostic markers of infection in patients with AKI.

### Design

We performed a prospective observational study between April 2013 and April 2014.

### Setting

Guy’s & St Thomas’ NHS Foundation Hospital is a tertiary care centre with a 43-bed, level 3 multi-disciplinary adult intensive care unit (ICU).

### Patient population

We recruited critically ill adult patients (≥18 years) with AKI stage 2 or 3 as per serum creatinine criteria of the Kidney Disease Improving Global Outcome classification [13]. The lowest serum creatinine in the preceding 12 months prior to hospitalisation was used as a baseline value; if not available, patients were excluded. Other exclusion criteria were: AKI stage 2/3 present for more than 36 h, pre-existing dialysis dependent renal failure, or an expected life expectancy < 48 h. We also excluded patients in whom serial blood sampling was not desirable (ie. Jehovah’s witness, patients with haemoglobin < 70 g/L).

### Collection of samples

Samples for serum suPAR were collected at baseline and 22-26 h, 46-50 h, 94-98 h and 142-146 h after enrolment. Serum CRP was measured daily throughout the 6-day study period. In patients receiving CRRT, effluent samples were collected at the same time points for measurement of suPAR. All samples were processed and stored in dedicated research freezers at -80 °C until batch analysis at the end of the study.

### Collection of clinical data

We collected baseline demographics, Acute Physiology and Chronic Health Evaluation (APACHE) II score and Sequential Organ Failure Assessment (SOFA) score on admission to ICU. Patients were categorised as having an infection if they had documented clinical signs combined with positive microbiology or imaging results supporting the diagnosis of an infection.

### Laboratory analyses

Serial suPAR concentrations were measured by enzyme-linked immunosorbent assay (ELISA) using the commercially available suPARnostic® ELISA Kit manufactured by ViroGates, Denmark. Samples were tested in singlets with inter-plate variation of 6%. CRP was measured using a latex enhanced immunoturbidimetric method on the Siemens Advia 2400 [analytical range: 4–336 mg/L and reference range: < 10 mg/L].

### Statistics

Patient characteristics, suPAR and CRP levels were summarised as frequency (percentage), mean [standard deviation (SD)] or median [interquartile range (IQR)], as appropriate. The relationship between suPAR and CRP at each time point was explored using Pearson correlation. Additionally, the correlations between suPAR and previous day CRP, CRP and previous day suPAR, and changes of suPAR and CRP from their respective previous measurement were calculated.

We compared the characteristics of patients who did and did not develop infections using the Chi-squared, Fisher’s exact and t-tests, as approriate. SuPAR and CRP levels of both groups were compared using Mann-Whitney tests, and areas under the receiver operating characteristics curves (AUC) were calculated to assess the ability of suPAR and CRP to discriminate between patients with an established infection and those who did not develop an infection during the study period.

## Results

We analysed the data of 55 patients with AKI stage 2 or 3 of whom 43 received CRRT during the 6-day study period. The mean age was 60.5 (15.9) years and 61.8% were male (Table 1). The majority were caucasian (60%), followed by Afro-Carribean (16.4%) and Asian (10.9%).

**Table 1:** Baseline demographics

Parameter	Total cohort (*n* = 55)
Age [years], mean (SD)	60.5 (15.9)
Male gender, n (%)	34 (61.8%)
Ethnicity	
Caucasian	60%
Afro-Caribbean	16.4%
Asian	10.9%
Other	12.7%
Diabetes Mellitus	18 (32.7%)
Any CKD	19 (34.6%)
CKD stages	
1	0
2	2 (3.6%)
3a	7 (12.7%)
3b	7 (12.7%)
4	2 (3.6%)
5	1 (1.8%)
ICU admission diagnosis, n (%)	
Post-major surgery	19 (34.5%)
Sepsis	16 (29%)
Respiratory failure	6 (10.9%)
Vasculitis	2 (3.6%)
Multi-organ failure	4 (7.2%)
Neurological emergency	2 (3.6%)
Cardiac arrest	1 (1.8%)
Other	5 (9.1%)
Severity of illness on admission to ICU	
APACHE II score, mean (SD)	19.4 (5.2)
SOFA score, mean (SD)	8.1 (2.4)

*Abbreviations*: *APACHE* Acute Physiology and Chronic Health Evaluation, *CKD* chronic kidney disease, *SD* standard deviation, *SOFA* sequential organ failure assessment, *ICU* Intensive care unit

### Biomarker results

**Fig. 1:**
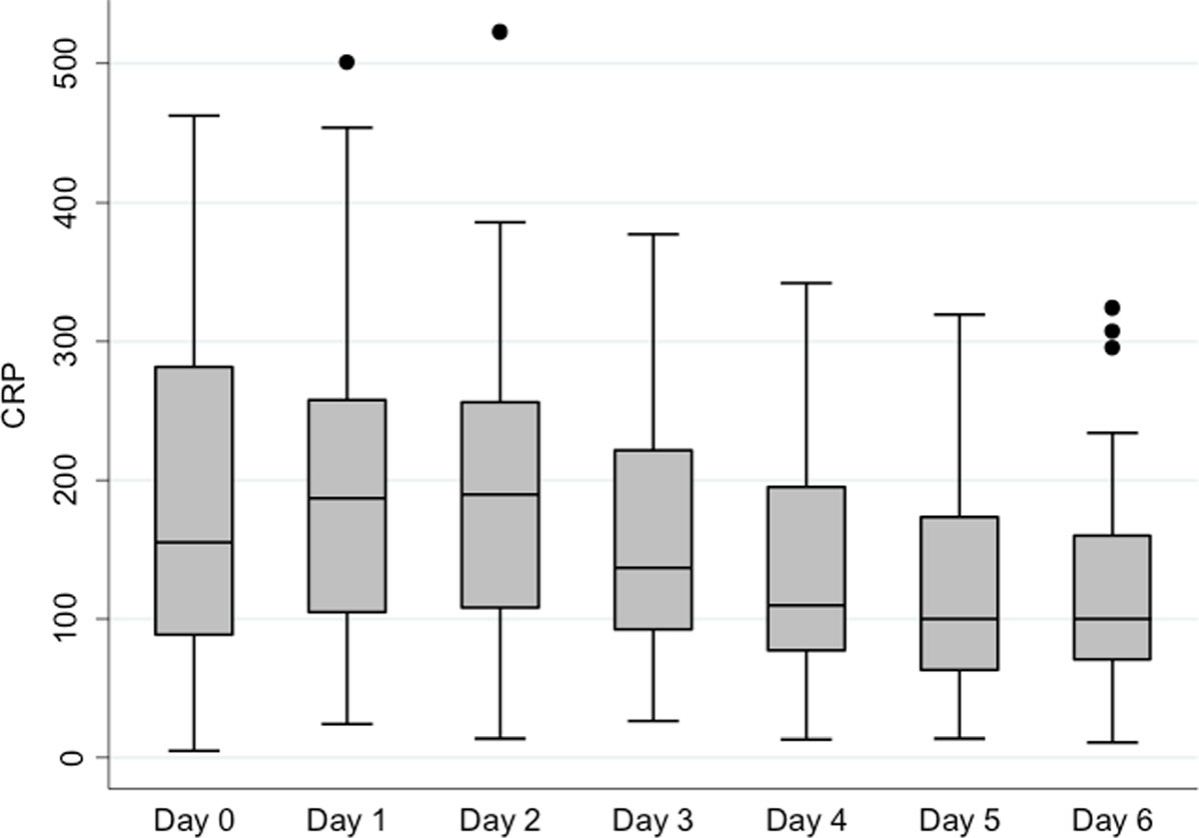
Serial serum CRP concentrations. Abbreviations: CRP = C-reactive protein

**Fig. 2:**
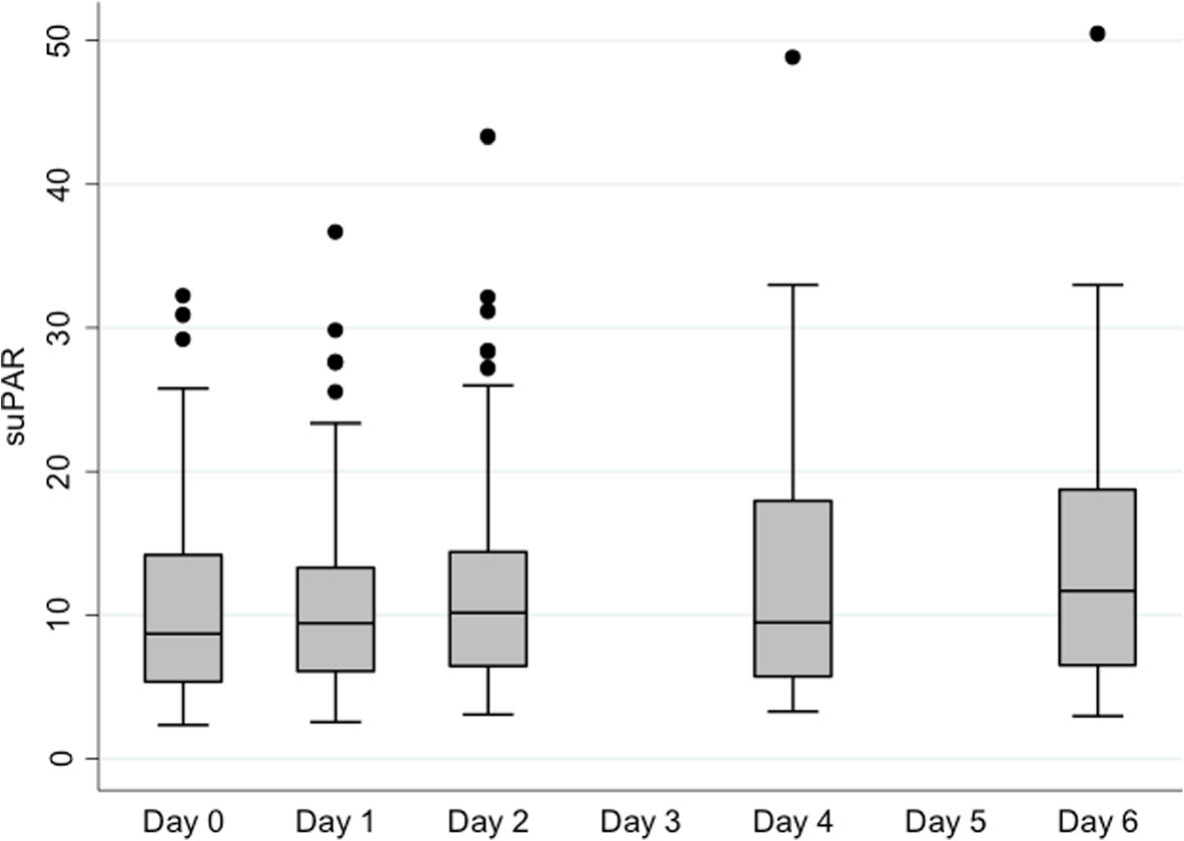
Serial systemic suPAR concentrations. Abbreviations: suPAR = soluble urokinase-type plasminogen activator receptor

Mean CRP levels increased between day of recruitment and day 1 and then declined from day 3 onwards (Table 2 and Fig. 1). In contrast, the systemic suPAR levels tended to remain stable or increase slighty across the 6-day study period (Table 2 and Fig. 2). In patients treated with CRRT, suPAR was not detectable in the effluent fluid.

**Table 2:** Correlations between mean systemic suPAR and CRP values

	suPAR [ng/ml] mean (SD)	CRP [mg/L] mean (SD)	Correlation
Day of enrolment	11.0 (7.6)	176.0 (113.2)	*r* = 0.234 *p* = 0.095
Day 1	11.7 (7.7)	199.5 (106.0)	*r* = 0.092 *p* = 0.519
Day 2	12.5 (8.9)	195.5 (105.6)	*r* = 0.018 *p* = 0.897
Day 3	–	160.2 (88.5)	
Day 4	12.6 (9.0)	142.3 (88.0)	*r* = 0.012 *p* = 0.935
Day 5	–	126.1 (80.6)	
Day 6	13.4 (9.0)	115.6 (74.2)	*r* = 0.039 *p* = 0..801

*Abbreviations*: *suPAR* soluble urokinase-type plasminogen activator receptor, *CRP* C-reactive protein, *r* regression, *SD* standard deviation

### Correlation between suPAR and CRP

**Fig. 3:**
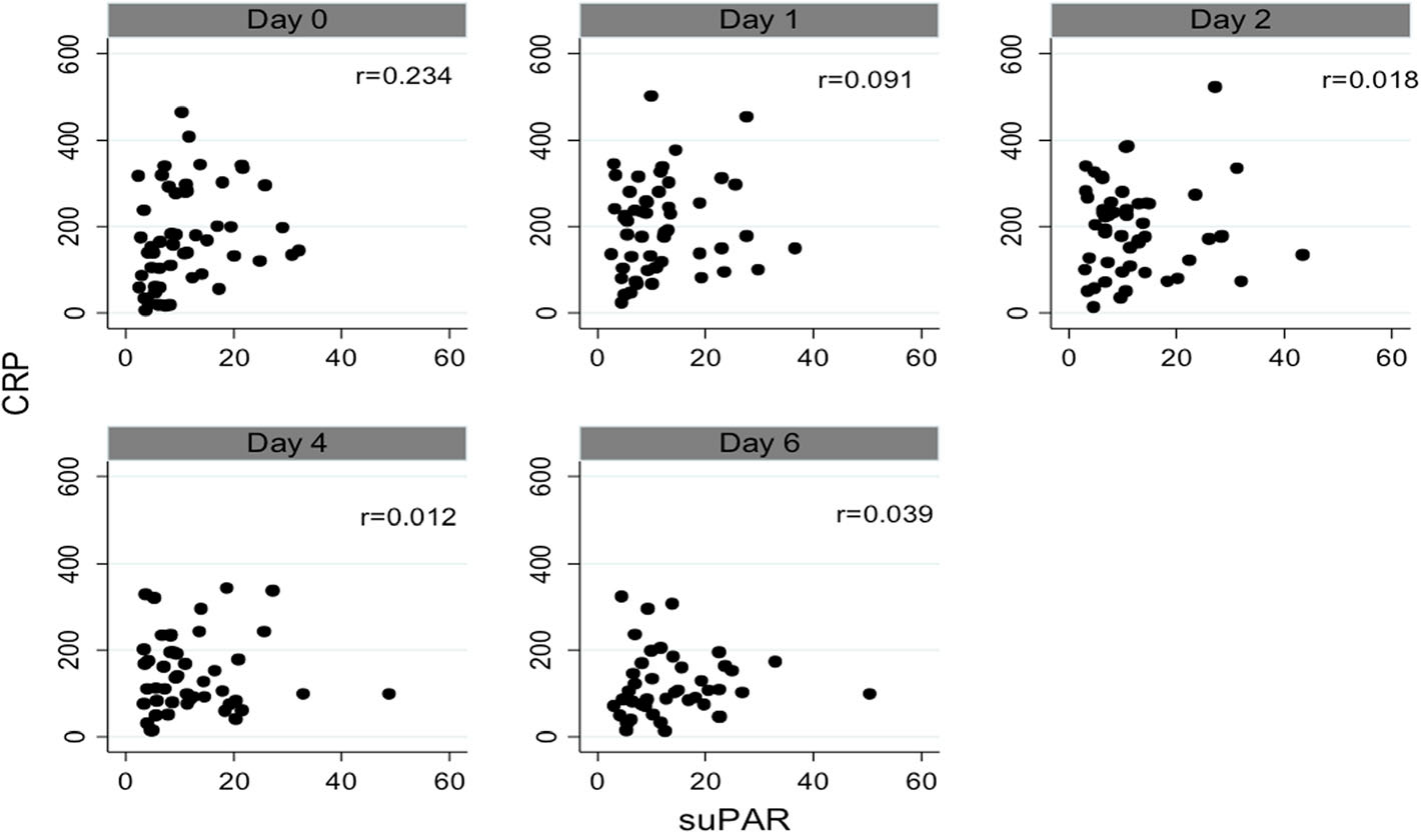
Scatter plots of daily suPAR and CRP values. Abbreviations: suPAR = soluble urokinase-type plasminogen activator receptor in ng/ml; CRP = C-reactive protein in mg/L

On the day of enrolment (ie. day 0), there was a weak to moderate correlation between systemic suPAR and CRP concentrations (*r* = 0.234) but this was not statistically significant (*p* = 0.095) (Table 2 and Fig. 3). After day 0, there was no significant correlation between CRP and suPAR results.

To allow for the possibility that CRP or suPAR concentrations changed at different rates, the correlations between values on consecutive days were calculated. No significant correlation between CRP and suPAR concentrations was detected (Table 3). However, when evaluating the change in suPAR and CRP values from the respective previous measurement, a weak correlation between change in suPAR and CRP from day 0 to day 1 (*r* = 0.298, *p* = 0.037) and day 1 to day 2 (*r* = 0.310, *p* = 0.029) was noted (Table 3). There was no correlation at any later time point.

**Table 3:** Correlations between systemic suPAR and CRP values on different days

	Correlation between suPAR result and previous day CRP value	Correlation between CRP value and previous day suPAR result	Correlation between change in suPAR and change in CRP values
Day 1	*r* = 0.228 *p* = 0.108	*r* = 0.042 *p* = 0.767	*r* = 0.298 *p* = 0.037
Day 2	*r* = − 0.026 *p* = 0.856	*r* = 0.090 *p* = 0.518	*r* = 0.310 *p* = 0.029
Day 3	–	*r* = − 0.104 *p* = 0.491	–
Day 4	*r* = − 0.049 *p* = 0.747	–	*r* = 0.232 *p* = 0.129
Day 5	–	*r* = 0.036 *p* = 0.819	–
Day 6	*r* = 0.048 *p* = 0.761	–	*r* = −0.166 *p* = 0.299

*Abbreviations*: *suPAR* soluble urokinase-type plasminogen activator receptor, *CRP* C-reactive protein, *r* regression

### Role of suPAR and CRP as markers of infection

There were no significant baseline differences between patients with and without an infection during the 6-day study period (Table 4). Values of suPAR were significantly higher in those with an infection across all time points but there was no difference in CRP results (Table 5). In patients with an infection, the respective AUCs of suPAR on the day of infection were higher than those of CRP values (Table 6).

**Table 4:** Characteristics of patients with and without infection

Parameter	No infection during 6-day study period *n* = 19	Infection during 6-day study period *n* = 36	*p*-value
Age, mean (SD)	61.5 (16.7)	60.0 (15.7)	0.75
Male sex, n (%)	12 (63.2)	22 (31.1)	0.88
Ethnicity, n (%)			
Caucasian	11 (57.9)	22 (61.1)	0.69
Afro-Carribean	4 (21.1)	5 (13.9)	
Asian	1 (5.3)	5 (13.9)	
Other	3 (15.8)	4 (11.1)	
BMI, mean (SD)	27.4 (5.5)	29.2 (8.9)	0.46
SOFA score on admission to ICU, median (IQR)	8 (7–10)	8 (7–9)	0.43
Diabetes Mellitus	4 (21.1)	14 (38.9)	0.23
Any CKD	7 (36.8)	12 (33.3)	0.99

*Abbreviations*: *BMI* body mass index, *ICU* intensive care unit, *IQR* interquartile range, *SD* standard deviation

**Table 5:** Comparison of suPAR and CRP values in patients with and without infections

	suPAR [ng/ml]					CRP [mg/L]				
	No infection during 6 day study period		Infection during 6 day study period		*p*-value	No infection during 6 day study period		Infection during 6 day study period		*p*-value
	n	Median (IQR)	n	Median (IQR)		n	Median (IQR)	n	Median (IQR)	
Day of enrolment	19	6.2 (4.2–9.2)	36	11.0 (6.5–16.1)	0.02	16	124 (60–238)	36	162 (105–286)	0.50
Day 1	19	7.2 (4.5–10.1)	35	11.4 (7.1–14.5	0.02	18	181 (80–241)	35	191 (118–280)	0.33
Day 2	18	8.1 (3.9–11.3)	34	10.8 (6.8–14.6)	0.04	19	171 (51–282)	35	204 (121–254)	0.49
Day 3						16	125 (67–212)	32	174 (98–227)	0.32
Day 4	17	6.7 (4.1–10.6)	34	11.7 (8.2–18.5)	0.02	17	135 (74–202)	31	109 (82–178)	0.94
Day 5						15	98 (46–196)	30	105 (63–169)	0.85
Day 6	18	8.4 (5.3–14.1)	30	12.6 (8.2–22.4)	0.04	15	89 (71–184)	32	101 (61–153)	0.65

*Abbreviations*: *suPAR* soluble urokinase-type plasminogen activator receptor, *CRP* C-reactive protein, *IQR* interquartile range

**Table 6:** Area under the receiver operating curves of suPAR and CRP for diagnosing an established infection

	suPAR			CRP		
	n	AUC	95% CI	n	AUC	95% CI
Day 0	43	0.73	0.57–0.89	40	0.58	0.39–0.77
Day 1	42	0.72	0.56–0.89	41	0.58	0.40–0.77
Day 2	44	0.69	0.52–0.56	45	0.54	0.35–0.73
Day 3				42	0.58	0.38–0.77
Day 4	47	0.71	0.55–0.87	45	0.48	0.29–0.68
Day 5				42	0.49	0.29–0.68
Day 6	48	0.674	0.51–0.83	47	0.46	0.27–0.65

*Abbreviations*: *suPAR* soluble urokinase-type plasminogen activator receptor, *CRP* C-reactive protein, *CI* confidence interval, *AUC* area under receiver operating characteristics curve

### Role of suPAR and CRP as predictors of infection

The majority of infections were diagnosed before of shortly after admission to the ICU. Thirty six patients had an infection during the study period of whom 5 (13.9%) were diagnosed prior to enrolment and 13 (36%) on day of enrolment; the remaining 18 patients (50%) were diagnosed later. Following exclusion of patients with an infection before or on day of ICU admission, the AUC of suPAR on day 0 for predicting an infection later was 0.62 (95% CI 0.43–0.80) compared to 0.50 (0.29–0.71) for CRP.

## Discussion

Our results show that CRP was a poor marker of infection in patients with severe AKI whereas suPAR had a stronger association with the development of an infection. We also confirmed that serum suPAR was not removed during CRRT.

Infections are the major causes of morbidity and mortality in patients with AKI [14]. Accurate and timely diagnosis is essential to enable clinicians to initiate appropriate and effective antimicrobial therapy early. The tools used by clinicians, namely clinical signs, inflammatory markers and imaging techniques, have important limitations [15].

SuPAR has emerged as a potential sepsis biomarker [2, 5, 6]. Under normal physiological conditions, suPAR is predominantly expressed by neutrophils, monocytes, macrophages and activated T-cells, and its serum concentration is relatively stable throughout the day [9]. In healthy adults, mean suPAR concentrations of 2000 pg/ml have been reported [16]. SuPAR has a molecular mass of approximately 55-60 kDa. While the membrane-bound uPAR appears to facilitate phagocytosis of bacteria, suPAR has chemotactic properties and facilitates recruitment of neutrophils and monocytes [8, 11, 17, 18]. Apart from infections, serum concentrations of suPAR may also be increased during inflammatory conditions, such as arthritis, cancer and liver disease [9]. Not surprisingly, several studies have shown that critically ill patients with systemic inflammatory response syndrome (SIRS), bacteraemia or sepsis had significantly higher suPAR results than healthy controls but the accuracy of suPAR to differentiate sepsis from SIRS was low [5, 12, 19–22].

Data on the role of suPAR in patients with renal disease are limited to patients with specific types of chronic kidney disease (CKD) like focal segmental glomerulosclerosis, diabetic nephropathy and lupus nephritis where plasma suPAR appears to have a pathological role resulting in proteinuria and renal scarring [23–26]. In a recent prospective observational trial in 107 consecutive elective cardiac surgery patients, suPAR levels were predictive of the development of AKI after surgery after exclusion of patients with pre-existing CKD [27]. Finally, in patients with cardiovascular risk factors, suPAR levels have been found to be associated with a more rapid decline in glomerular filtration rate [23].

This is the first study that focused on the role of suPAR as a marker of infection in patients with severe AKI. The main result was that suPAR only had moderate sensitivity and specificity but performed better than CRP. This is an important finding since CRP is often used in clinical practice as an aid to diagnose and monitor the course of an infection. We acknowledge that further research is necessary to confirm these findings. It will also be important to compare the performance of suPAR with that of procalcitonin (PCT), and to evaluate the role of suPAR in combination with existing severity of illness scores.

We acknowledge other potential limitations. First, as a single centre study, there is reduced generalisability of the results to centres with a different patient-case mix. Second, we categorised patients as having an infection based on a retrospective review of all clinical data, laboratory results and microbiological reports. It is possible that patients with an infection were missed or mis-classified. Third, suPAR concentrations can be affected by other inflammatory conditions, cancer and liver disease. In our analysis, we did not control for these potential confounders. Fourth, we only enrolled patients with AKI stage 2 and 3 and did not distinguish between different types and aetiologies of AKI, and it is possible that the performance of suPAR varies depending on the aetiology or stage of AKI. Finally, our sample size was relatively small. Larger studies are necessary to validate our findings, to determine a diagnostic cut-off of suPAR in patients with AKI and to explore whether suPAR levels correlate with the clinical course following an infection.

## Conclusions

Our study showed that suPAR concentrations had better diagnostic and predictive value than CRP to detect infections in critically ill patients with severe AKI. Larger studies are necessary to confirm our findings.

## Abbreviations

AKI: Acute kidney injury
APACHE: Acute Physiology and Chronic Health Evaluation
AUC: Area under receiver operating characteristics curve
BMI: Body mass index
CI: Confidence interval
CKD: Chronic kidney disease
CRP: C-reactive protein
CRRT: Continuous renal replacement therapy
ELISA: Enzyme-linked immunosorbent assay
ICU: Intensive care unit
IQR: Interquartile range
PCT: Procalcitonin
REC: Research Ethics Committee
RRT: Renal replacement therapy
SD: Standard deviation
SIRS: Systemic inflammatory response syndrome
SOFA: Sequential organ failure assessment
suPAR: Soluble urokinase-type plasminogen activator receptor
uPAR: Urokinase plasminogen activator receptor
